# Comparison of linkage and association mapping in MAGIC lines identifies *AtMTP3* as a new gene controlling natural variation in leaf zinc concentration in Arabidopsis

**DOI:** 10.1093/jxb/eraf142

**Published:** 2025-03-31

**Authors:** Felipe Klein Ricachenevsky, Ana Carolina A L Campos, Paloma Koprovski Menguer, Fernando Mateus Michelon Betin, Jaime Tovar, William F A van Dijk, Mary Lou Guerinot, David E Salt, Paula X Kover

**Affiliations:** Center for Biotechnology, Federal University of Rio Grande do Sul, Porto Alegre, RS, Brazil; Botany Department, Institute of Biosciences, Federal University of Rio Grande do Sul, Porto Alegre, RS, Brazil; Rijk Zwaan, Phytopathology Research, 2678 ZG De Lier, The Netherlands; Institute of Biological and Environmental Sciences, University of Aberdeen, Cruickshank Building, Aberdeen AB24 3UU, UK; Center for Biotechnology, Federal University of Rio Grande do Sul, Porto Alegre, RS, Brazil; Center for Biotechnology, Federal University of Rio Grande do Sul, Porto Alegre, RS, Brazil; Wellcome Genome Campus, Hinxton, Cambridgeshire CB10 1SA, UK; Institute of Biological and Environmental Sciences, University of Aberdeen, Cruickshank Building, Aberdeen AB24 3UU, UK; PBL Netherlands Environmental Assessment Agency, 2500 GH The Hague, Netherlands; Department of Biological Sciences, Dartmouth College, Hanover, NH 03755, USA; Institute of Biological and Environmental Sciences, University of Aberdeen, Cruickshank Building, Aberdeen AB24 3UU, UK; School of Biosciences, University of Nottingham, Sutton Bonington, UK; Milner Centre, University of Bath, Bath BA2 7AY, UK; CIMMYT, Mexico

**Keywords:** AtMTP3, genome-wide association, ionome, quantitative trait loci, zinc

## Abstract

The *Arabidopsis thaliana* MAGIC lines are the result of extensive recombination among 19 accessions, which allows a direct comparison of association and linkage mapping using the same population. We used both approaches to map the genetic basis of natural variation in the leaf ionome of *A. thaliana*. We found 57 quantitative trait loci (QTL) and 10 significant associations, eight of which co-locate with QTL analysis. This suggests that the genome-wide association has a low rate of false positives in these MAGIC lines, but an overall lower power to identify potential genetic factors explaining natural variation. We replicated several loci previously identified by linkage or association studies and identified new candidate genes. We demonstrated the success of this approach by validating AtMTP3 (a vacuolar zinc and cobalt transporter) as the cause of natural variation in zinc leaf concentration. We showed that Kn-0, one of the MAGIC lines founder accessions, carries a rare *AtMTP3* allele that results in increased zinc concentration in leaves. Yeast mutant complementation suggests that Kn-0 *AtMTP3* encodes a hypofunctional protein compared with Col-0. Our work demonstrates that natural variation in Zn leaf concentration is linked to vacuolar transport and Zn sequestration in roots, opening up new avenues to manipulate Zn concentration in plants.

## Teaser

We used multiparent mapping lines to compare linkage and association mapping and found good complementarity and power, leading to the identification of a new control for natural variation in shoot zinc concentration.

## Introduction

Plants exhibit extensive natural phenotypic variation for several traits including adaptive and economically important ones such as flowering time, growth, disease resistance, tolerance to drought, and elemental composition. Understanding the genetic basis of this natural variation has been a major focus of crop improvement and is key to understanding plant adaptation. Because these traits are usually complex and affected by multiple loci and environmental factors, the identification of the genetic basis of these traits has been quite challenging. The main approach to identify loci underlying natural variation in complex plant traits is to associate genotypic and phenotypic variation using either linkage analysis in recombinant inbred lines (RILs), also known as ‘QTL analysis’, or a genome-wide association study (GWAS) in a collection of natural accessions (Jaganathan *et al.*, 2020).

The advantages of using GWAS in a large worldwide population are that through a long history of mutation and recombination, the linkage disequilibrium between common variants decays down to a few kilobases (Kim *et al.*, 2007). Thus, a single nucleotide polymorphism (SNP) association with a complex trait is likely to be very close to the causal variant, and GWAS can offer superior localization of candidate causal variants. The disadvantage is that population structure, epistasis, and natural selection can cause indirect associations, and a high rate of false positives (John *et al.*, 2022). The number of false positives can be improved by controlling for population structure, although such an approach can also reduce the power to detect associations (Liu *et al.*, 2016). The power of GWAS to detect associations with rare variants is more limited compared with linkage analysis, especially if they do not account for much of the phenotypic variation (John *et al.*, 2022).

Mapping lines for linkage analysis have been typically produced by crossing two inbred accessions (genomes independently collected from natural populations) showing contrasting phenotypes for the trait of interest, and are mainly the result of inbreeding an F_2_ population (e.g. Hu *et al.*, 2020; Shariatipour *et al.*, 2021). Due to the limited number of recombination events, these lines typically do not provide good mapping accuracy, with QTL being localized to chromosomal regions varying between 5 cM and 50 cM (which is equivalent to 1.2–12 Mb in *Arabidopsis thaliana* and 9–90 Mb in maize) (Alonso-Blanco *et al.*, 2005). The development of multiparent RILs, such as the Multiparent Advanced Genetic InterCross (MAGIC) lines (Cavanagh *et al.*, 2008), has improved the power of linkage mapping. Mapping lines derived from intercrossing multiple accessions for multiple generations provide increased numbers of recombination events, and a larger number of segregating alleles. As a result, QTL can be mapped to significantly smaller regions (<1 Mb) (Kover *et al.*, 2009; Camargo *et al.*, 2018; Han *et al.*, 2020) and the relationship between multiple traits can easily be studied within the same set of mapping lines.

Linkage mapping and GWAS have complementary characteristics: in linkage mapping lines, the minor allele frequency is not lower than 1/[no. of of parental founders], and population structure is broken up. However, these lines show higher linkage disequilibrium, causing reduced mapping resolution. GWAS can provide a more precise localization of the causal gene but can also lead to the detection of more false positives. While GWAS can be used in MAGIC populations, it is expected to have reduced power compared with a large set of accessions due to a limited number of alleles entering the analysis. It has been suggested that combining linkage mapping and GWAS to map the same trait simultaneously would be best (Bergelson and Roux, 2010; Zhang *et al.*, 2019). However, the direct comparison of the two approaches using equivalent genetic material has not yet been performed.

The ionome is defined as the inorganic composition of an organism (Salt *et al.*, 2008). Ionomics is an approach in which high-throughput multielemental quantification is combined with genomics to understand the genetic basis of the ionome. In *A. thaliana*, several studies have used either linkage analysis or GWAS to identify loci associated with natural variation in elemental concentrations (reviewed by Huang and Salt, 2016). The common approach is to quantify the elemental composition of plant tissues such as leaves and seeds in natural accessions, which are next used for GWAS. Alternatively, accessions with extreme elemental concentrations can be crossed to generate populations for QTL mapping. QTL analysis, GWAS, and bulk segregant analysis (BSA) have been used for mapping causative genes for natural variation in sodium (Baxter *et al.*, 2010), cobalt (Morrissey *et al.*, 2009), arsenic (Chao *et al.*, 2014b; Sánchez-Bermejo *et al.*, 2014), cadmium (Chao *et al.*, 2012), molybdenum (Tomatsu *et al.*, 2007; Baxter *et al.*, 2008), sulfur (Loudet *et al.*, 2007; Koprivova *et al.*, 2013; Chao *et al.*, 2014a), selenium (Chao *et al.*, 2014a), and zinc (Zn;Chen *et al.*, 2018; Pita-Barbosa *et al.*, 2019b). However, much of the genetic basis of ionomic variation in natural accessions of *A. thaliana* remains to be uncovered, which would be useful to identify candidate genes for natural variation in other economically important plant species (Whitt *et al.*, 2020).

Here, we use the ionome of the *A. thaliana* MAGIC lines to make a direct comparison of the QTL and GWAS mapping strategies using the same set of genotypes. We show that these lines are a powerful resource for identifying the genetic basis of natural variation. We demonstrate this by the identification of several new candidate genes with ionomic phenotypes. Additionally, we characterize a new allele of a gene previously not known to be related to natural variation in Zn (*AtMTP3*).

## Materials and methods

### Plant phenotyping for QTL mapping

#### Plant materials and growth conditions

Ionomic experiments were performed using the *A. thaliana* MAGIC population developed by Kover *et al.* (2009) and a collection of natural accessions (as controls across trays). Seeds for all the MAGIC lines used here, for the 19 parental accessions of the MAGIC lines (Col-0, Bur-0, Ct-1, Edi-0, Hi-0, Kn-0, Ler-0, Mt-0, No-0, Po-0, Oy-0, Rsch-4, Sf-2, Tsu-0, Wil-2, Ws-0, Wu-0, and Zu-0) and for the accessions HKT2.4, ICE107, ICE163, and Koch-1 (used to characterize *AtMTP3*), were obtained from the European Arabidopsis Stock Centre (NASC).

Plants were grown for 4 weeks in a controlled growth room with 10 h days (100 µmol photons m^–2^ s^–1^)/14 h nights and a temperature of ~25 °C during the day and 17 °C at night . Each plant was grown in 32 mm 7C Jiffy peat soil pellets evenly distributed in trays with 104 cells lined with capillary matting. Before sowing, the 104 Jiffy soil pellets of one tray were soaked with 2.6 litres of solution containing subtoxic concentrations of arsenic, cadmium, cobalt, lithium, nickel, selenium, strontium, and rubidium. The final concentration of these elements in dry soil were: 23.4 mg kg^–1^ Na_2_HAsO_4_·7H_2_O; 0.55 mg kg^–1^ Cd(NO_3_)_2_·4H_2_O; 5.82 mg kg^–1^ Co(NO_3_)_2_·6H_2_O; 13.78 mg kg^–1^ LiNO_3_; 5.81 mg kg^–1^ Ni(NO_3_)_2_·6H_2_O; 5.52 mg kg^–1^ K_2_SeO_4_; 21.16 mg kg^–1^ Sr(NO_3_)_2_, and 20.35 mg kg^–1^ RbNO_3_. Seeds of each accession/line were sown in the pre-soaked Jiffy pellets and stratified at 4 °C for 1 week. Plants were bottom-watered three times (days 10, 17, and 24) with 1/4 strength Hoagland nutrient solution [1.5 mM KNO_3_, 1 mM Ca(NO_3_)_2_·4H_2_O, 0.5 mM MgSO_4_·7H_2_O, 0.25 mM NH_4_H_2_PO_4_, 11.5 µM H_2_BO_3_, 1.3 µM MnCl_2_·4H_2_O, 0.2 µM ZnSO_4_·7H_2_O, 0.075 µM CuSO_4_·5H_2_O, 0.028 µM MoO_3_) in which Fe was replaced by 10 µM Fe-HBED. Trays were rotated every day to help reduce gradient effects of light, temperature, and humidity. After 4 weeks, 2 leaves (2–5 mg DW) of each plant were harvested, washed with 18 MΩcm Milli-Q water, and placed in Pyrex tubes for subsequent digestion.

#### Phenotyping elemental concentration

Leaf samples were harvested into Pyrex tubes and dried for 20 h at 88 °C. For every tray, the dry weight of seven reference samples was measured and used to calculate the weights and final concentration of the elements of the remaining samples based on a heuristic algorithm which uses the best-measure elements in these samples as described in Lahner *et al.* (2003). Sample processing and ionomic profiling analyses were conducted as described in Campos *et al.* (2021). A liquid reference material composed of pooled digested leaf samples of the first 10 trays was prepared and analysed after every nine samples to correct for variation within inductively coupled plasma-MS (ICP-MS) analysis runs (Danku *et al.*, 2013). The calibration standards (with indium internal standard and blanks) were prepared from single element standards solutions.

Plants were grown in a pipeline fashion in which every day one tray was sown and one tray was harvested, as described in Campos *et al.* (2021). Each tray had 104 plants randomly distributed of which 28 were MAGIC lines which are used in this experiment, and 76 *A. thaliana* accessions that were used in a separate study (Campos *et al.*, 2021). In total, 120 trays were grown over a period of 37 weeks, yielding six replicates of each accession/line. Values of elemental concentrations greater than the 90th percentile+2×(90th–10th percentile) or lower than the 10th percentile–2×(90th–10th percentile) were considered extreme outliers and excluded. Remaining negative values were set to zero. To normalize between trays and ICP-MS runs, we used a linear mixed model with tray and ICP-MS run as fixed effects and accession/line as random effect. We estimated elemental levels for each line using the best linear unbiased prediction (BLUP) for each line and element. BLUP values were estimated using the package lme4 in R (Bates *et al.*, 2015) with line, tray, ICP-MS run, and replicate as variables, and are shown in Supplementary Table S1.

### Linkage and association mapping

MAGIC lines were used for trait mapping using both a linkage and an association approach. The linkage analysis was performed using 1536 SNP markers and the HAPPY package as described in Kover *et al.* (2009). For this approach, we used the 512 MAGIC lines listed in Supplementary Table S1.

The association analysis was performed using the software GAPIT (Lipka *et al.*, 2012; https://zzlab.net/GAPIT/) and a subset of the SNPs identified in Imprialou *et al.* (2017). Previous studies found no evidence of genetic structure among the MAGIC lines (Kover *et al*., 2009;  Gan *et al.*, 2011). We also use GAPIT to identify potential population structure and found no evidence (see kinship matrix, Supplementary Fig. S1). Thus, we chose not to use any correction for population structure during the association analysis. All genomic short read sequence data for MAGIC lines used here are available from ENA under study accession number PRJEB19252. The selection criteria for SNPs to be included in this study were that they were bi-allelic, and that there were no more than 50% of the MAGIC lines with missing data, yielding a total of 1,993,755 SNPs (accessible at https://doi.org/10.5281/zenodo.15305277). We performed a GWAS using the 392 MAGIC lines listed in Supplementary Table S1 that have been sequenced and phenotyped. For a fairer comparison between the linkage and GWAS approaches, we repeated the HAPPY analysis with only the 392 MAGIC lines used for GWAS.

### Functional characterization of *AtMTP3* natural variation

#### *AtMTP3* sequence and ionomics data analyses

To identify all known AtMTP3 haplotypes within *A. thaliana* sequence diversity, we downloaded all AtMTP3 predicted protein sequences from the 1001 Arabidopsis genomes website (https://1001genomes.org/). Sequences were downloaded using the browser http://signal.salk.edu/atg1001/3.0/gebrowser.php and aligned using MEGA (Tamura *et al.*, 2021). Haplotype groups are listed in Supplementary Tables S2 and S3.

In order to analyse which mutations in the Kn-0 sequence were the best candidate for being causal of the phenotype observed, we compared ionomic profiling data from different experiments. These data were used to compare Col-0 and Kn-0 Zn leaf concentration (trays 778, 1489, and 1539) and to compare Col-0, Kn-0, Koch-1, HKT2.4, ICE107, and ICE163 Zn leaf concentration (trays 1818–1825) (Supplementary Table S4).

#### Yeast phenotype complementation

The coding sequence of *AtMTP3.2* described as being functional (Arrivault *et al.*, 2006) was cloned in the pDR195 vector using the *Xho*I and *Bam*HI sites and specific primers (Supplementary Table S5). Constructs were transformed into *Saccharomyces cerevisiae* using the lithium acetate/polyethylene glycol (LiAc/PEG) method (Gietz and Schiestl, 2007). Yeast strains used were: wild-type BY4741 (MATa, *his3****-****1*, *leu2****-****0*, *met15****-****0*, *ura3 0*); and the *zrc1 cot1* double mutant (MATa; *his3-1*, *leu2-0*, *met15-0*, *ura3-0*, zrc1::natMX cot1::kanMX4) for Zn and Co complementation. Complementation tests were performed as described in Menguer *et al.* (2013) and Ricachenevsky *et al.* (2018), with variations in Zn and Co concentration added.

#### Plant phenotype complementation

Plant phenotype complementation was conducted by inserting the full-length *AtMTP3* genomic locus from Col-0 plants into Kn-0 plants. The sequence spanning the *AtMTP3* locus (3951 bp) was amplified from Col-0 genomic DNA and cloned into pCAMBIA1300 using specific primers (Supplementary Table S5) and the *Eco*RI and *Bam*HI sites. The plant expression construct carrying Col-0 *AtMTP3* driven by its native promoter was transformed into *Agrobacterium tumefaciens* EHA105 by electroporation. The floral-dip method (Clough and Bent, 1998) was used to transform Kn-0 plants with the addition of 100 μM acetosyringone to the culture 3 h before dipping to induce *vir* genes. Homozygous T_3_ plants were screened on plates containing half-strength Murashige and Skoog medium supplemented with 0.5% w/v sucrose, 0.8% w/v phytagel, and 50 mg l^–1^ kanamycin.

#### Gene expression analysis

Plant experiments under axenic conditions were performed in square plastic Petri dishes (12 cm×12 cm). Plants were kept in a controlled-environment growth room with a day/night cycle of 16 h light/8 h dark, 24 °C, and 130 µmol m^–2^ s^–1^ of photosynthetically active radiation (PAR). For gene expression, sterile *A. thaliana* seeds from Kn-0 and Col-0 accessions were plated on control full-strength B5 medium with 0.8% w/v phytagel or medium with modified concentrations of Fe and Zn. The treatments included 200 µM and 400 µM ZnSO_4_, and Fe depletion (no Fe added+0.153 mM ferrozine). Seeds were stratified at 4 °C for 48 h prior to transfer to a controlled-environment growth room. Plates were incubated vertically for 15 d, and root samples were harvested for RNA extraction.

Total RNA was extracted from root tissues, frozen in liquid nitrogen, and stored at –80 °C using the Direct-zol™ RNA Miniprep system according to the manufacturer’s instructions. First-strand cDNA synthesis was performed with oligo(dT) and reverse transcriptase (M-MLV, Invitrogen^®^) using 1 μg of RNA. Quantitative real-time PCR analysis (qPCR) was carried out in a StepOne™ Real-Time PCR System (Applied Biosystems), and SYBR® Green I (Thermo Fisher Scientific) was used as fluorescent reporter dye. The *A. thaliana* gene ubiquitin 10 (UBQ10, AT4G05320) was used as the reference gene for normalization. All primers used for gene expression analyses are listed in Supplementary Table S5. Data were analysed using the 2^–∆∆Ct^ method described by Livak and Schmittgen (2001).

## Results

### Mapping ionomic trait loci with known causal genes

To compare the efficacy of linkage and association approaches, we started by mapping two phenotypes with known causal genes that have been previously phenotyped in MAGIC lines (Kover *et al.*, 2009): the ‘ERECTA’ and ‘GLABROUS’ phenotypes. ERECTA is known to be caused by a transversion of a T to an A in amino acid 750 of the protein expressed from the *ERECTA* gene (AT2G26330; chromosome 2: base pairs 11,208,183–11,213,971) present only in the Ler-0 accession (Torii *et al.*, 1996). With the linkage analysis, we found a peak on chromosome 2: base pair 11,300,373, and correctly identified that the haplotype from Ler is the only one to confer the phenotype (Supplementary Fig. S2). Searching 250 kb above and below the peak, we can identify 100 candidate genes. However, assuming that the phenotypic change is caused by a non-synonymous substitution exclusive to Ler, we can narrow it down to three potential candidate genes, one of which is *AT2G26330*. In comparison, when we used an association approach, we found a single peak with a clear association with the *ERECTA* gene (peak located at the SNP ER_472: base pair 11,208,543).

Among the MAGIC lines, *GLABROUS* is known to be caused by a deletion in the coding sequence of the *GL1* gene (AT3G27920; chromosome 3: base pairs 10,361,945–10,363,506) derived from the parental accession Wil-2 (Hauser *et al.*, 2001). The linkage analysis identifies a strong peak 104 bp away from the causal locus (Supplementary Fig. S3). However, without previous knowledge of the causal gene, and assuming that the phenotype is due to a non-synonymous substitution unique to Wil-2 within a ±250 kb interval around the peak, we would have selected 26 genes, none of which is the known causal gene. Thus, this approach could lead to failure in the identification of causal loci. Alternatively, if we look for differentially expressed genes between Wil-2 and the other 18 accessions on the same interval using the data from Gan *et al.* (2011), we find that 37 out of 93 genes in the interval have at least one pairwise significant difference in expression. Three genes have most of the significant difference between Wil-2 and the other accessions: *AT3G27610*, *AT3G27830*, and *AT3G27920* (GL1). Thus, this approach would have significantly helped in narrowing down the number of candidate genes. The GWAS approach identifies a cloud of 104 SNPs equally associated on chromosome 3 between 10,111 kb and 10,330 kb, which is located 31 kb forward of the causal gene. Thus, in this case, a GWAS approach does not provide good localization of the causal gene. This is perhaps not surprising since indels are not included in the GWAS polymorphism dataset, a fact often underappreciated.

### Mapping genes responsible for variation in elemental concentrations using linkage and association methods

**Fig. 1.**
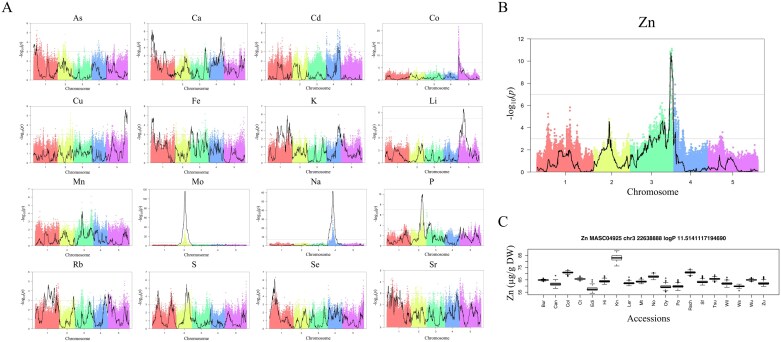
Quantitative trait loci (QTL) identified with linkage and association approaches using the leaf ionomics profile dataset of MAGIC lines. (A) The results for each element tested. The results for the association analysis are shown with points of different colours for associations in each chromosome. The linkage results are overlayed on top, with a continuous black line. (B) The results for linkage and association for zinc in more detail, and (C) the phenotypic effects calculated for each haplotype under the linkage peak in chromosome 3.

Using linkage mapping, we identified 57 QTL for 18 of the elements measured (no QTL was detected for boron or nickel; Fig. 1A). Between one and nine QTL were detected per element (see Table 1 and Supplementary Table S6 for a detailed description of QTL found for each element). In contrast, using GWAS, we detected 10 significant associations, with a false discovery rate (FDR) <0.05 for six elements (Fig. 1A; Supplementary Table S7). Only two out of the 10 associations identified with the GWAS approach were not observed using the QTL approach. However, because there are more MAGIC lines that can be used in the QTL approach than with the GWAS approach, we also performed QTL mapping using the exact same set of 392 lines as the GWAS for a fairer comparison. With 392 MAGIC lines, we observed fewer QTL than with 512 lines: 37 QTL for 17 elements. Still, the QTL mapping identified more QTL than when using the association approach with the same lines, and included seven of the 10 associations identified with GWAS.

**Table 1.** List of elements measured in the experiment for which a significant QTL was detected

Element	No. of QTL identified (linkage)	Log_10_*P*	No. of QTL identified (association)	QTL not replicated with 392 lines	Known genes for natural variation	Distance from QTL peak (kb)
Arsenic	2	3.8	0	1	*HAC1* (AT2G21045)	Not detected
Cadmium	1	3.6	0	0	*HMA3* (AT4G30120)	58
Calcium	6	6.2	0	3		
Cobalt	2	9.9	1	0	*FPN2* (AT5G03570)	278
Copper	2	5.6	0	1		
Iron	2	4.8	0	1	*IRT1* (AT4G19690)	Not detected
Lithium	1	8.6	0	0		
Magnesium	4	10	1	1		
Manganese	1	4.2	0	1	*NRAMP1* (AT1G80830)	Not detected
Molybdenum	2	120	1	0	*MOT1;1* (AT2G25680)	140
Phosphorus	5	10	1	3		
Potassium	7	5.8	0	2		
Rubidium	9	4.7	0	3		
Selenium	1	6.1	0	0	*APR2* (AT1G62180)	Not detected
Sodium	1	61	1	0	*HKT1* (AT4G10310)	128
Strontium	2	3.6	0	1		
Sulfur	5	5.5	0	1	*APR2* (AT1G62180) and *ATPS1* (AT3G22890)	1352 and not detected
Zinc	4	10.7	3	2	*HMA4* (AT2G19110)	150

The second column shows results using 512 MAGIC lines and linkage analysis; log_10_*P* indicates the highest likelihood detected for each element. We compared the overlap of results using GWA in the fourth column. The fifth column indicates the number of QTL observed with 512 lines that were not replicated when only the 392 lines used for GWA were used in the linkage analysis. We also list the genes already known to affect the natural variation in these elements and their distance to the observed peak using the linkage approach.

Ten genes that explain natural variation in elements were previously identified (see Table 1). The linkage mapping with 512 and 392 MAGIC lines identifies peaks that co-locate with six and five of these genes, respectively (see Table 1 and Supplementary Table S6). In contrast, the GWAS analysis identified only three associations that co-located with these previously known genes (Supplementary Table S7). In all three cases, however, the GWAS associations were located closer to the causal gene than the peak identified with the linkage approach. For example, the QTL peak for sodium is 128 kb from *AtHKT1*, a well-known gene that underlies natural variation in sodium accumulation (Busoms *et al.*, 2018). The GWAS analysis also identifies a clear peak at the same location, but the strongest association is located 8 kb away from *AtHKT1*. These results suggest that the GWAS approach is less powerful but more accurate in localization, even when used in MAGIC lines.

Locations identified with all three approaches were typically the QTL with higher heritability (see Supplementary Table S7); *R*^2^ for these genes varied from 0.09 to 0.56, while QTL genes observed only with the 512 lines had heritability values varying from 0.055 to 0.095 (see Supplementary Table S5). The three genes with the highest heritability are well supported by the three approaches, and the top two have well-characterized genes (*AtMOT1* for Mo and *AtHKT1* for Na; Fig. 1A) explaining their function in *A. thaliana*. Thus, we decided to pursue the genetic basis under the association explaining the third highest heritability, which is the QTL for variation in zinc (Zn) concentration on chromosome 3 (Fig. 1B).

### Identifying a new candidate gene for natural variation in zinc accumulation

A QTL for variation in Zn concentration, located at base pair 22,130,442 on chromosome 3, was also supported by a peak of significant association centred at base pair 22,141,418 using GWAS (Fig. 1B). At this location, we compared the Zn leaf concentration of MAGIC lines that have the 19 parental haplotypes, and determined that the high accumulation of Zn is determined by the Kn-0 haplotype (Fig. 1C). This inference is supported by the fact that Kn-0 is in the top 3% of plants with the highest Zn concentration (108.46 µg g^–1^) out of 1135 accessions analysed by Campos *et al.* (2021). Col-0, one of the MAGIC population parental accessions and the most used reference accession for *A. thaliana* studies, has a Zn leaf concentration close to the population average (90.02 µg g^–1^). Thus, we used Col-0 as our reference accession for comparisons with Kn-0 in order to identify the gene responsible for the high Zn concentrations in the leaves.

Searching for possible candidate genes in the regions identified by linkage and GWAS, we identified *AtMTP3* (*AT3G58810*), which encodes a known Zn/Co vacuolar transporter that is up-regulated in roots under high Zn, high Mn, or Fe deficiency conditions, and has a role in detoxifying excessive Zn entering roots by sequestering it in vacuoles (Arrivault *et al.*, 2006). *AtMTP3* silencing resulted in increased Zn concentration in leaves, presumably due to decreased Zn partitioning into root vacuoles and a resultant increased Zn root-to-shoot transport (Arrivault *et al.*, 2006). Therefore, we hypothesized that Kn-0 has a non-functional, hypofunctional, or low-expression allele of *AtMTP3*, which would result in increased Zn accumulation in leaves.

**Fig. 2.**
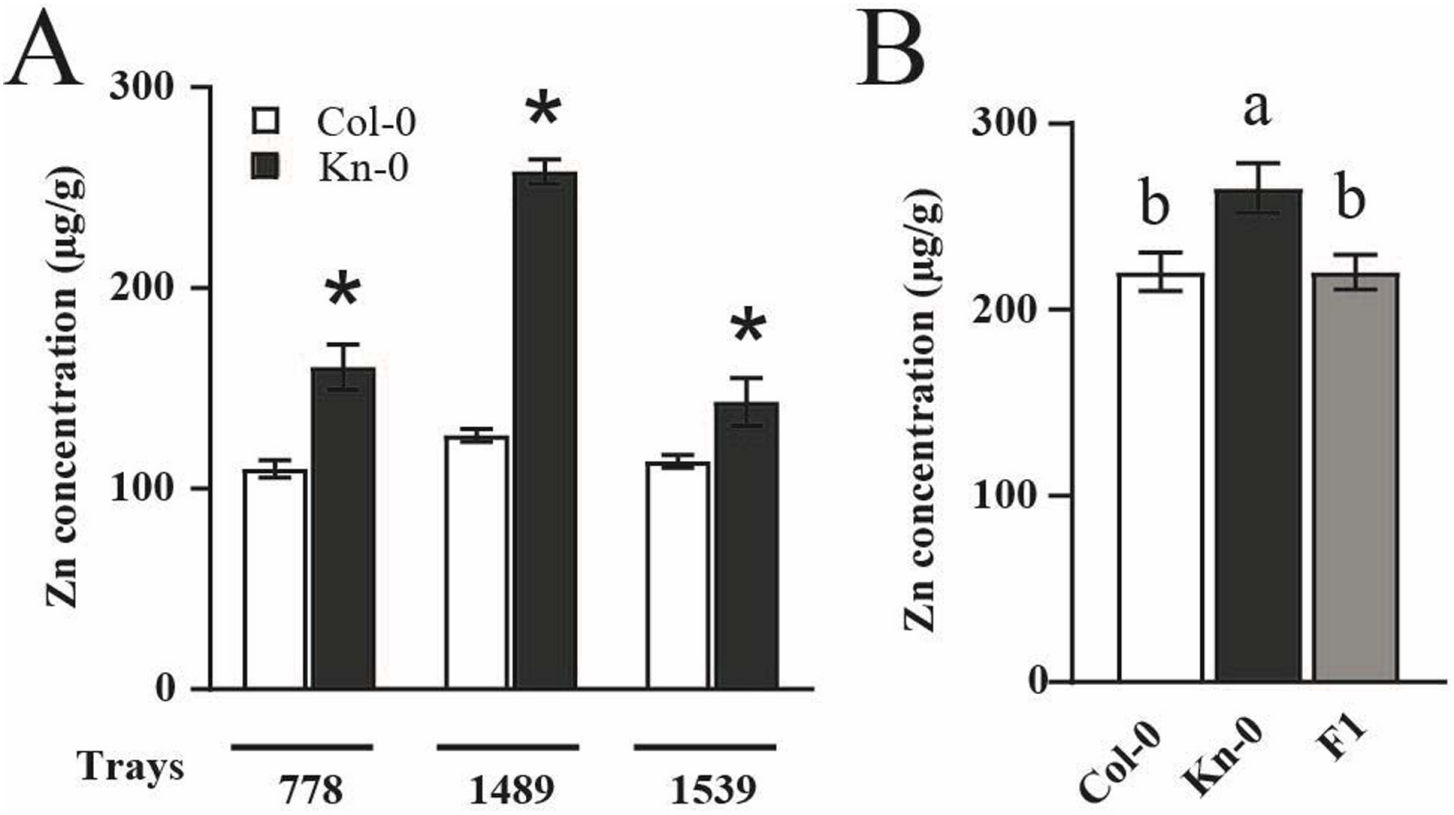
High Zn concentration in leaves in Kn-0 compared with Col-0 is a recessive phenotype. (A) Leaf Zn concentration of Col-0 and Kn-0 plants from three independent experiments (performed in distinct laboratories, by different experimenters, in similar growth room conditions). Asterisks indicate significant differences comparing means using *t*-test, *P*≤0.05. Number of replicates: tray 778 (*n*=24); tray 1489 (*n*=12); tray 1539 (*n*=12). (B) Zn concentration in leaves of Col-0 and Kn-0 plants, and F_1_ plants from a cross of Col-0×Kn-0 parentals. Different letters indicate significant differences using one-way ANOVA and Tukey HSD, *P*≤ 0.05. Number of replicates: Col-0 (*n*=8); Kn-0 (*n*=8); F_1_ Col-0×Kn-0 (*n*=16).

To validate this hypothesis, we analysed three independent experiments comparing leaf Zn concentrations of Col-0 and Kn-0 (Supplementary Table S4). The results show that Kn-0 has a 26–103% higher Zn concentration compared with Col-0 (Fig. 2A). To investigate the inheritance pattern of this variation in phenotype, we crossed the two accessions, and quantified Zn in leaves of Col-0, Kn-0, and Col-0×Kn-0 F_1_ plants. The F_1_ plants had Zn concentrations similar to Col-0, indicating that the Col-0 allele is dominant over the high accumulating Kn-0 allele (Fig. 2B). This shows that the Kn-0 allele at the causal locus is a recessive allele.

### *AtMTP3* is the causal gene for natural variation in zinc concentration in leaves of *A. thaliana*

**Fig. 3.**
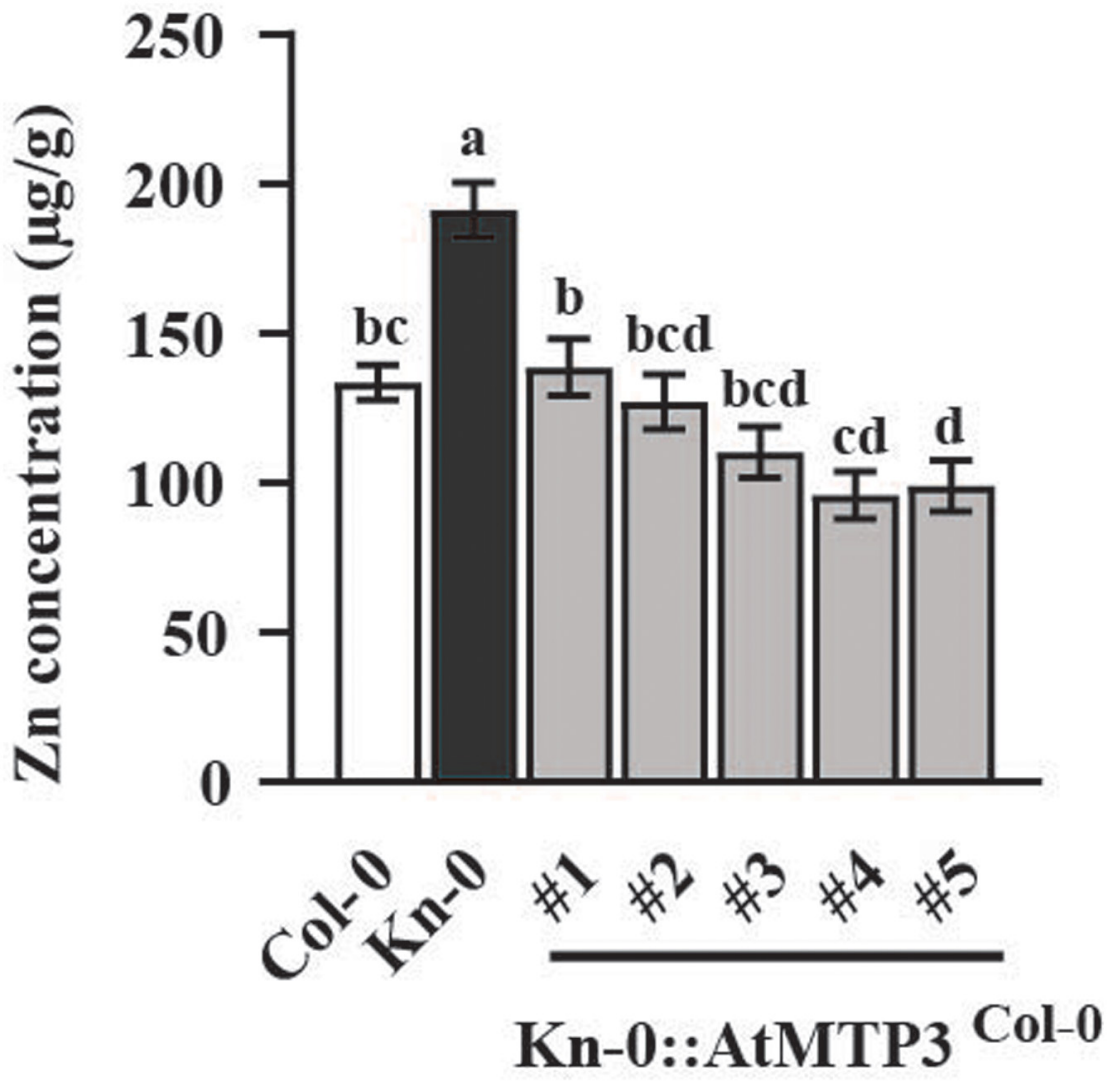
*AtMTP3* is the causal gene for the Kn-0 high Zn leaf concentration phenotype. Leaf Zn concentration of Col-0 and Kn-0 plants and five Kn-0 transgenic lines complemented with the Col-0 *AtMTP3* genomic locus. Different letters indicate significant differences using one-way ANOVA and Tukey HSD, *P*≤ 0.05; *n*=8.

To confirm that *AtMTP3* is the causal gene for the observed variation in Zn concentration, we complemented Kn-0 plants with the full genomic region of *AtMTP3* from Col-0 to generate Kn-0:*AtMTP3*^Col-0^ plants. We cultivated Col-0, Kn-0 wild-type plants, and Kn-0:*AtMTP3*^Col-0^ complemented plants in soil and quantified Zn concentration in leaves. The results showed that Kn-0:*AtMTP3*^Col-0^ lines had a Zn leaf concentration similar to Col-0, whereas Kn-0 showed a higher Zn concentration in the same experiment (Fig. 3). These results support the conclusion that *AtMTP3* is the causal gene for the elevated Zn concentrations in Kn-0 leaves.

**Fig. 4.**
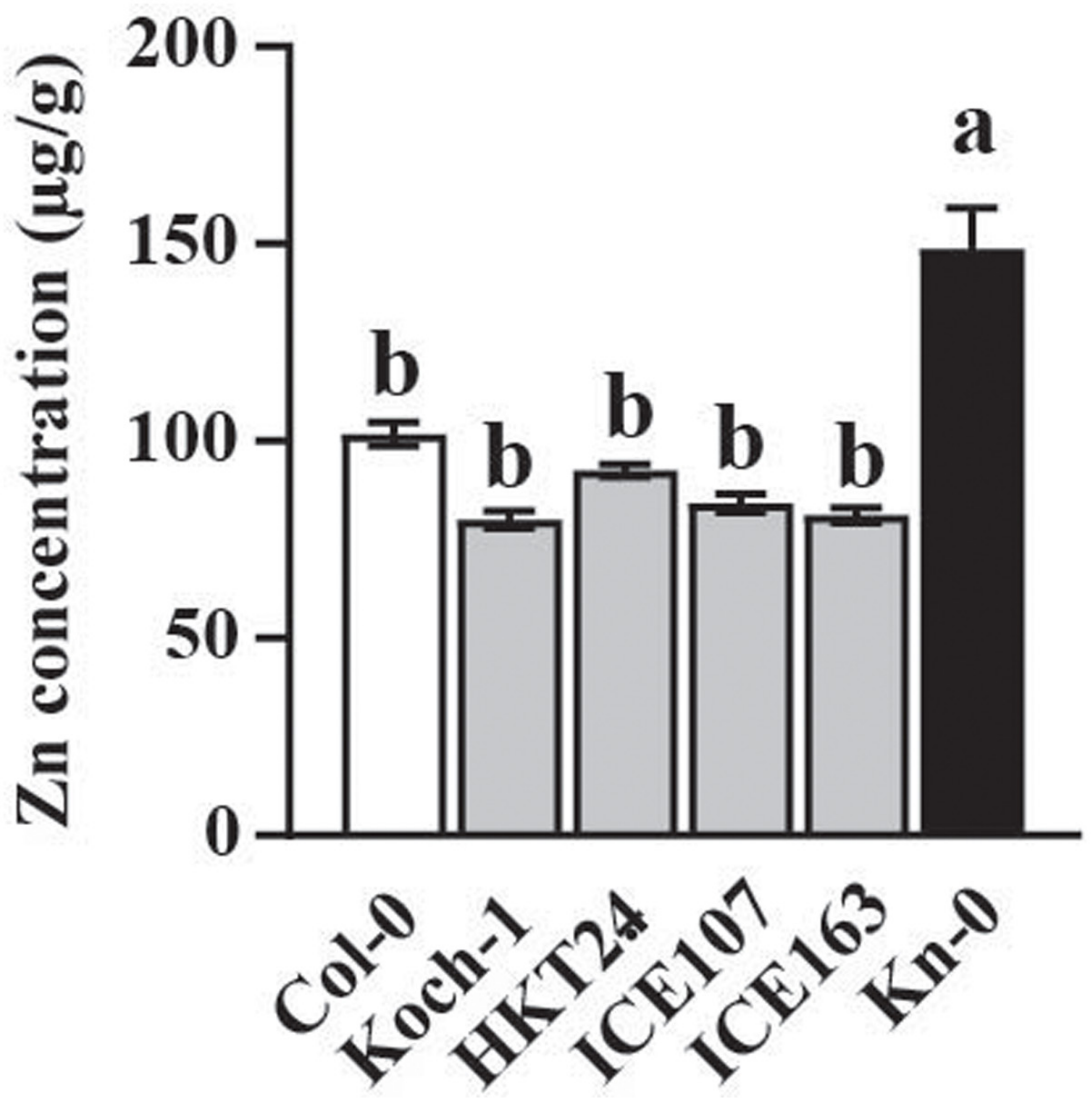
Kn-0 has a unique SNP in the *AtMTP3* locus that is likely to be causal for the leaf high Zn phenotype. Leaf Zn concentration of Col-0, Koch-1, HKT24, ICE107, ICE163, and Kn-0 plants (*n*=8–12; different letters indicate significant differences using one-way ANOVA and Tukey HSD, *P*≤0.05). Number of replicates: Col-0 (*n*=8); Koch-1 (*n*=8); HKT2.4 (*n*=8); ICE107 (*n*=8); ICE163 (*n*=8); Kn-0 (*n*=12).

Since the MAGIC lines that inherited the Kn-0 haplotype showed higher Zn concentrations, we reasoned that the causative mutation should be exclusive to Kn-0 among the MAGIC founder accessions. Therefore, if *AtMTP3* is the causative gene, the Kn-0 genome should contain polymorphisms near the *AtMTP3* locus that are absent in the other 18 MAGIC founder accessions. We identified six SNPs meeting these criteria, ranging from base pair 21,747,168 to 21,751,533 in chromosome 3 (corresponding to base pair –2798 to +1568, considering the predicted transcriptional start site of *AtMTP3* at base pair 21,749,966 in chromosome 3; Table 2). To identify the most likely causal mutation, we examined four *A. thaliana* accessions that shared various combinations of these SNPs: HKT2.4, ICE 107, ICE 163, and Koch-1 (Table 2). Next, we compared the ionomic profiles of Kn-0, HKT2.4, ICE 107, ICE 163, and Koch-1 (Supplementary Table S4). As Col-0 was used as a reference in all experiments, Zn concentration values were normalized to Col-0. Clearly, the four accessions had Zn concentrations in their leaves similar to Col-0, while Kn-0 exhibited higher Zn concentrations (Fig. 4). Therefore, we concluded that the T-to-A polymorphism at base pair 21,751,533 is the most likely causative mutation. This mutation results in an amino acid substitution from isoleucine, a neutral amino acid, to lysine, a positively charged amino acid, in the AtMTP3 transmembrane domain 6, which may affect its transport function. We analysed the AtMTP3 protein sequences of 855 *A. thaliana* accessions and found 17 haplotype groups (Supplementary Tables S2, S3). Interestingly, the Kn-0 mutation is unique; that is, it is not shared with any other accession in *A. thaliana*.

**Table 2.** Polymorphisms exclusive to Kn-0 compared with other MAGIC lines founder accessions

Genomic position	Relative position	Accessions					
		Col-0	Kn-0	HKT2.4	ICE107	ICE163	Koch-1
21 747 168	–2798	G	A	A	A		A
21 747 381	–2585	T	A	A	A	A	A
21 747 899	–2067	C	A	A	A		A
21748 208	–1758	A	T	T			T
21 750 639^*a*^	674	C	A	A			A
21 751 533^*b*^	1568	T	A				

Accessions that share polymorphism with Kn-0 are shown. Polymorphisms that change coding sequence are highlighted.

^*a*^ T**C**T to T**A**T, serine to tyrosine.

^b^ A**T**A to A**A**A, isoleucine to lysine.

### Functional analysis of the *AtMTP3* allele from Kn-0

**Fig. 5.**
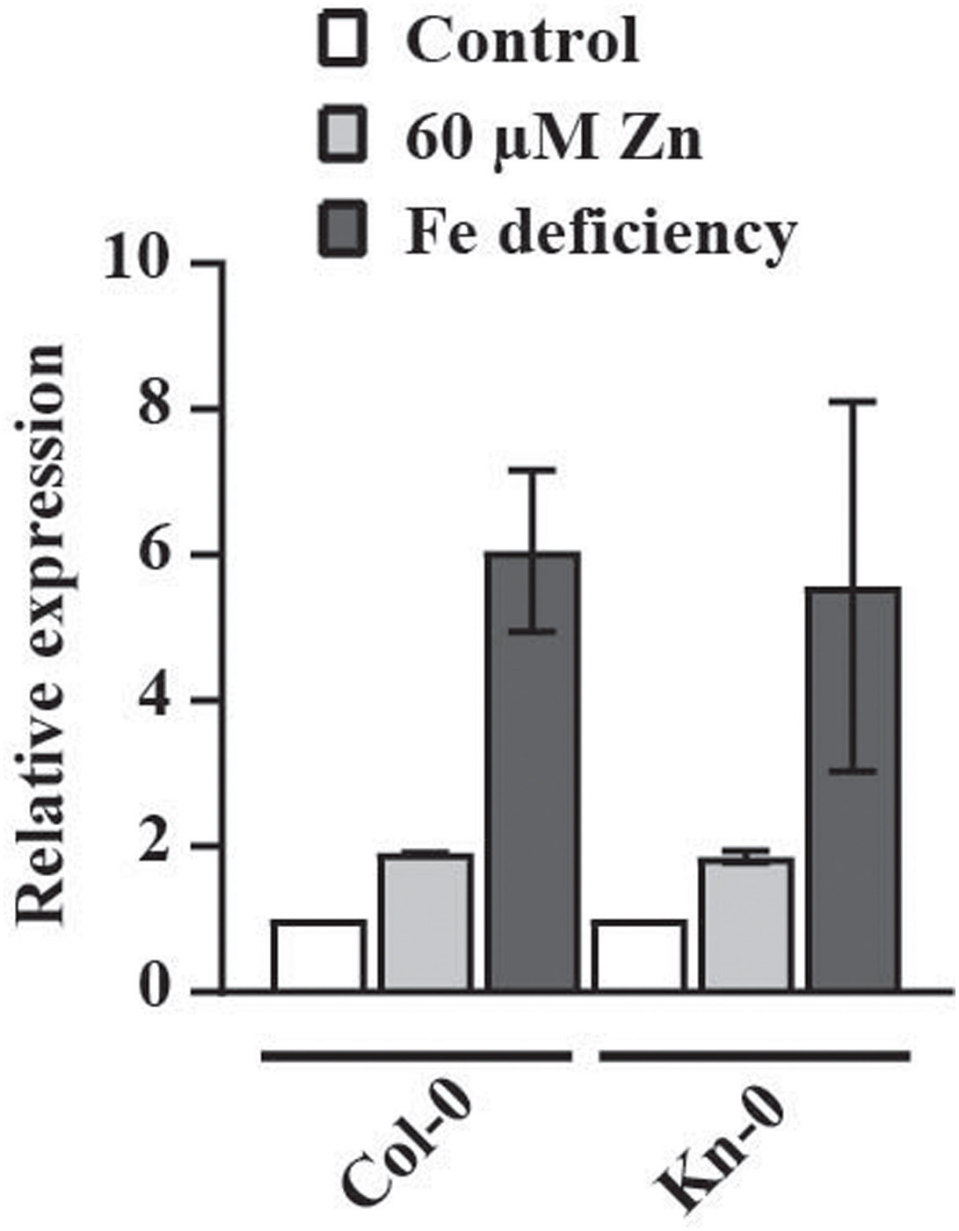
Col-0 and Kn-0 *AtMTP3* are up-regulated by Zn excess and Fe deficiency to a similar extent. *AtMTP3* gene expression analyses by RT-qPCR. Roots from 2-week-old Col-0 and Kn-0 plants grown under control conditions or exposed to Zn excess (60 µM) and –Fe (no Fe added) for 1 week were analyzed (*n*=4).

We conducted a functional study of the *AtMTP3* genes in Col-0 and Kn-0 to better understand their roles in these accessions. We hypothesized that the Kn-0 allele may exhibit lower expression than the Col-0 allele, resulting in reduced Zn partitioning into root vacuoles and consequently higher Zn concentrations in the leaves. We quantified *AtMTP3* gene expression in roots of plants from both accessions grown under Zn excess (60 µM Zn) and Fe deficiency (no Fe added), conditions which were shown to up-regulate *AtMTP3* expression (Arrivault *et al.*, 2006). Our results show that *AtMTP3* is up-regulated in roots by both treatments compared with the control, with similar levels of up-regulation in Kn-0 and Col-0 (Fig. 5). Thus, the higher accumulation of Zn observed in Kn-0 plants does not seem to be linked to lower expression of *AtMTP3*.

**Fig. 6.**
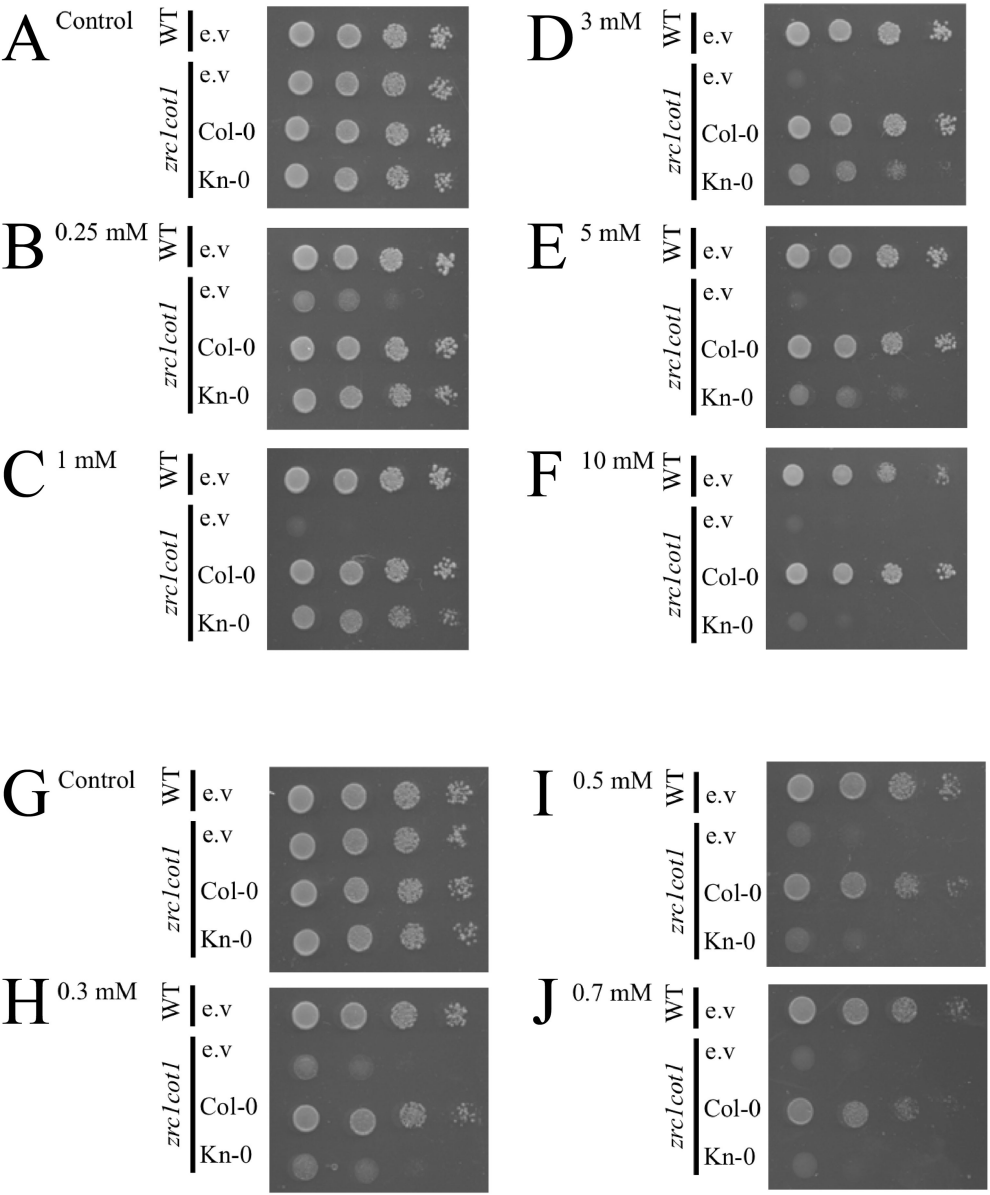
AtMTP3 protein from Kn-0 is hypofunctional from Zn and Co vacuolar detoxification. Yeast complementation assays using wild-type (WT) yeast and the *zrc1cot1* double mutant that is sensitive to Zn and Co excess due to lack of vacuolar detoxification. The WT and *zrc1cot1* were transformed with an empty vector (e.v.), and *zrc1cot1* was complemented with Col-0 or Kn-0 *AtMTP3* and grown in different Zn or Co concentrations in serial dilutions. (A) Control medium; (B) 0.25 mM Zn; (C) 1 mM Zn; (D) 3 mM Zn; (E) 5 mM Zn; (F) 10 mM Zn; (G) Control medium; (H) 0.3 mM Co; (I) 0.5 mM Co; (J) 0.7 mM Co.

Next, we performed yeast phenotypic complementation assays using the double mutant *zrc1cot1*, which lacks the ability to detoxify Zn and Co in the vacuole. We grew the wild-type yeast strain, *zrc1cot1* empty vector control, and *zrc1cot1* transformed with either *AtMTP3*^Col-0^ or *AtMTP3*^Kn-0^ under increasing Zn concentrations in the growth medium. At 0.25 mM Zn, both *AtMTP3*^Col-0^- and *AtMTP3*^Kn-0^-expressing *zrc1cot1* has similar growth compared with the wild type, while empty vector-transformed *zrc1cot1* yeast does not, indicating that proteins encoded by the *AtMTP3* alleles of Col-0 and Kn-0 are functional and able to detoxify Zn by transporting it into vacuoles (Fig. 6A, B). However, when the Zn concentration in the medium was increased to 1, 3, or 5 mM, *AtMTP3*^Col-0^-transformed *zrc1cot1* exhibited growth comparable with the wild-type strain, whereas *AtMTP3*^Kn-0^-transformed *zrc1cot1* was unable to grow (Fig. 6C–E). At 10 mM Zn, *AtMTP3*^Kn-0^-transformed *zrc1cot1* could not grow, whereas *AtMTP3*^Col-0^-transformed *zrc1cot1* growth was still comparable with that of the wild type (Fig. 6F). These results indicate that the *AtMTP3* allele from Kn-0 encodes a hypofunctional protein. Since AtMTP3 is also capable of Co transport (Arrivault *et al.*, 2006), we performed similar experiments with increasing Co concentrations in the growth medium. We found that the Kn-0 allele of *AtMTP3* is not able to suppress the Co sensitivity of the *zrc1cot1* mutant when cultivated in medium containing elevated levels of Co, whereas growth of *zrc1cot1* transformed with Col-0 *AtMTP3* was similar to that of the wild type (Fig. 6G–J). Thus, our results suggest that the AtMTP3 protein encoded by the Kn-0 allele is hypofunctional for Zn and Co transport.

## Discussion

The use of multiparental mapping lines to identify the genetic basis of complex traits in crops has increased in recent years (Scott *et al.*, 2020). Their power and usefulness stem from increased recombination and allelic diversity through the inclusion of multiple alleles, as well as specific germplasm that can be directly used for breeding. As demonstrated here (see Table 1), the *A. thaliana* lines produced by intermating 19 accessions are particularly powerful for identifying the genetic basis of complex traits. Using 512 MAGIC lines, we identified more QTL than any previous study on natural variation in elemental concentration using GWA or linkage alone (Baxter *et al.*, 2008, 2010; Morrissey *et al.*, 2009; Chao *et al.*, 2012; Forsberg *et al.*, 2015). In addition to identifying six out of the 10 previous known genes underlying natural variation in elemental concentration, we identified 51 other QTL; including one location which we could narrow down to a new causal gene for variation in Zn accumulation in a straightforward manner. The four loci already known to be linked to ionomic natural variation but not identified in this study are due to single rare alleles present in a single accession not included in the MAGIC lines: *AtHAC1* (Chao *et al.*, 2014b), *AtIRT1* (Tergemina *et al.*, 2022), *AtNRAMP1* (Tergemina *et al.*, 2022), and *AtATPS1* (Koprivova *et al.*, 2013). MAGIC lines will always miss such variants, but nevertheless it is impressive that the majority of the known loci could be detected, considering that they only include 19 parental accessions.

The resequencing of the parental lines (Gan *et al.*, 2011), together with imputation techniques (Imprialou *et al.*, 2017), has significantly improved the application of these lines for mapping studies, for example by allowing their use for GWAS and haplotype identification, and facilitating the identification of causal genes, as we show here in the case of *AtMTP3*. GWAS approaches have mainly focused on using collections of accessions (Sasaki *et al.*, 2022; Almira Casellas *et al.*, 2023), but GWAS has been successfully used in MAGIC populations of a few crops (Huang *et al.*, 2021). Here we used 392 MAGIC lines for GWAS and identified 10 potential associations (see Supplementary Table S7). GWAS using natural accessions is known for better localization but a high rate of false positives. Using MAGIC lines, we found that indeed the localization of known genes is often more accurate with GWAS than linkage, as demonstrated for *AtHKT1* (QTL peak ~1128 kb away; GWAS peak ~8 kb away). However, in some cases, as with the glabrous phenotype, it can be much worse. Since eight of the 10 associations observed were also present with QTL analysis, the rate of false positives does not seem to have been a big problem in these lines, but the power of detecting associations when compared with using linkage analysis on the exact same lines and phenotypes is limited. Nevertheless, GWAS in MAGIC lines was very successful in identifying QTL for elemental concentration when compared with GWAS using natural accessions. For example, using 349 accessions and GWAS, Chen *et al.* (2018) did not find any association with Zn natural variation. However, here, with only 392 MAGIC lines created from 19 accessions, we were able to find three associations. Since using MAGIC lines allows GWAS and QTL analysis, they may be more advantageous than using a collection of accessions, since they are also useful as starting points for creating near isogenic lines or as breeding material (Scott *et al.*, 2020).

Identifying candidate genes from QTL locations remains a challenge. Using traits for which we know the genetic basis of variation (i.e. *ERECTA* and *GLABROUS*), we show that there is no single approach that would be more successful. Nevertheless, we were successful in identifying a new causal gene for variation in Zn accumulation by leveraging the parental haplotype information and the sequenced data that exist for multiple *A. thaliana* accessions. Natural variation identified in *A. thaliana* has been used to identify candidate genes in other species, and in some cases to describe the mutations involved in them (Chao *et al.*, 2012; Pita-Barbosa *et al.*, 2019a; Sun *et al.*, 2019; Whitt *et al.*, 2020).

Two genes are known to control Zn natural variation in *A. thaliana*: *AtHMA4* and *AtHMA3* (Chen *et al.*, 2018; Pita-Barbosa *et al.*, 2019b). Our study shows that *AtMTP3* also affects natural variation in Zn accumulation in leaves. Arrivault *et al.* (2006) have previously shown the involvement of this gene with detoxification of Zn as a root vascular transporter. However, here we found a naturally occurring allele in the accession Kn-0 that encodes a hypofunctional version of AtMTP3, which under control conditions causes Zn accumulation in leaves. Silencing of *AtMTP3* resulted in increased Zn accumulation in leaves of *A. thaliana* (Arrivault *et al.*, 2006), which agrees with our results (see Fig. 6). We also did not find variation in *AtMTP3* expression (Fig. 5), indicating that protein-level variation is involved in the hypofunctionality of the Kn-0 *AtMTP3* allele, which could involve changes in protein stability, localization, transport, or regulatory function. Therefore, our work links *AtMTP3* allelic variation with Zn natural variation and provides a candidate gene for regulating the Zn concentrations of aerial tissues. Considering that homologous genes are responsible for natural variation in different plant species (Ren *et al.*, 2005; Baxter *et al.*, 2010; Chao *et al.*, 2012; Yan *et al.*, 2016; Pita-Barbosa *et al.*, 2019b), our findings could be the basis to find causal genes involved in controlling Zn levels in other plants species, including crops, with implications for biofortification. Moreover, our work opens up new questions about the ecological relevance of variation in root Zn sequestration, such as whether this variation has implications for local adaptation as well as in which environments higher Zn concentrations could be adaptive.

## Supplementary data

The following supplementary data are available at *JXB* online.

Fig. S1. The kinship matrix produced by GAPIT using the VanRaden method for the 392 MAGIC lines used in the GWAS.

Fig. S2. Plot combining LOD curves and Manhattan plots for the *ERECTA* phenotype.

Fig. S3. Plot combining LOD curves and Manhattan plots for the *GLABROUS* phenotype.

Table S1. List of BLUP values for the accumulated quantities for each element in each MAGIC line.

Table S2. Haplotypes for *AtMTP3* found in *Arabidopsis thaliana* genomes.

Table S3. *Arabidopsis thaliana* accessions grouped by *AtMTP3* haplotype.

Table S4. Data from ionomic profiling of leaves from different *Arabidopsis thaliana* accessions.

Table S5. Primers used in this work.

Table S6. List of all QTL detected with linkage, including position, Log*P*, *R*^2^ values, and SNP frequency.

Table S7. List of all significant associations detected with GAPIT, including *P*-values, *R*^2^, and SNP frequency.

eraf142_suppl_Supplementary_Tables_S1-S7

eraf142_suppl_Supplementary_Figures_S1-S3

## Data Availability

Data are available in the article and its supplementary data.
