# Supplementary material for: Comparison of linkage and association mapping in MAGIC lines identifies AtMTP3 as a new gene controlling natural variation in leaf zinc concentration in Arabidopsis
Source: J Exp Bot. 2025 Mar 31;76(14):3972–83. doi: 10.1093/jxb/eraf142 (PMC12448848; doi:10.1093/jxb/eraf142)
Supplement: eraf142_suppl_Supplementary_Figures_S1-S3 [file eraf142_suppl_supplementary_figures_s1-s3.pdf]

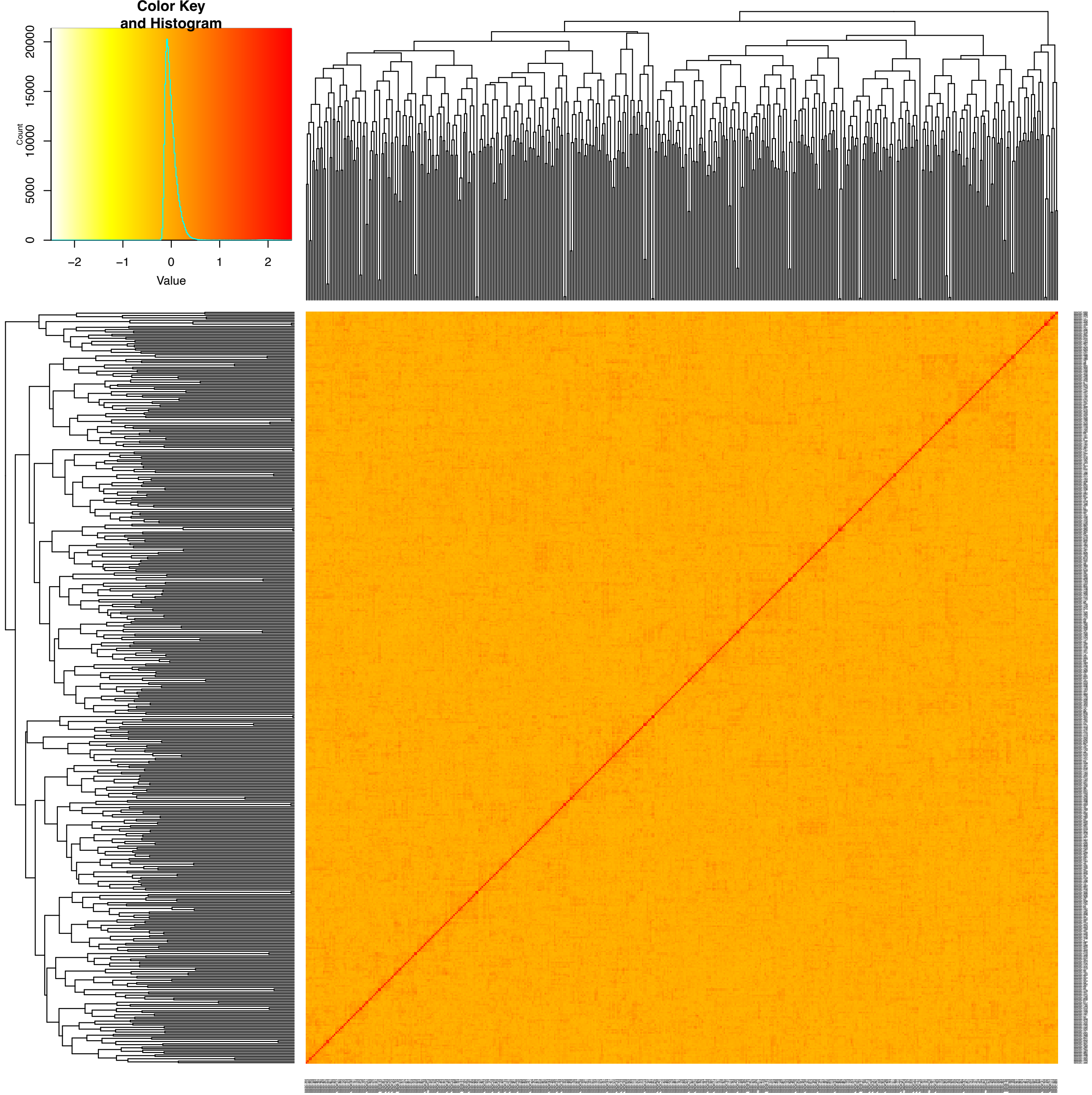

Figure S1. The kinship matrix produced by GAPIT using the VanRaden method for the 392 MAGIC lines used on the GWAS.

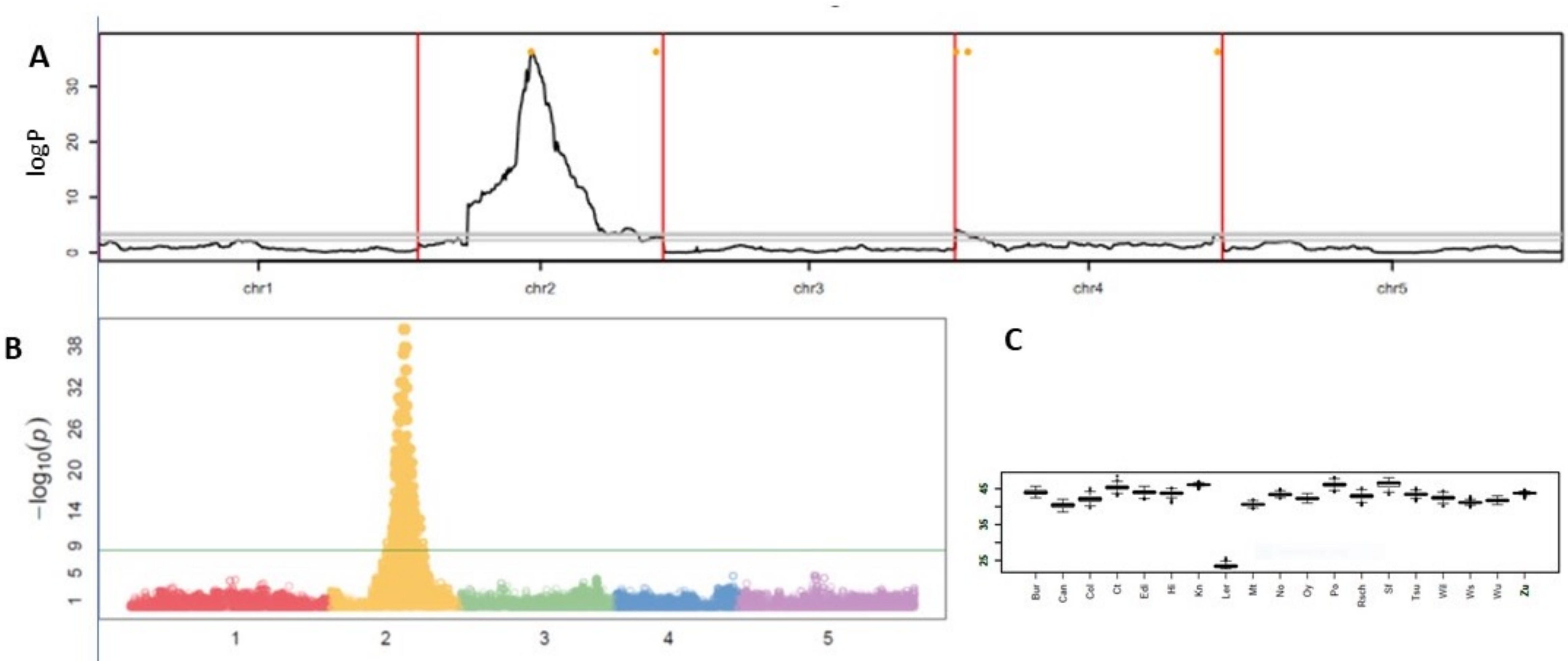

Figure S2. **Plot combining LOD curves and Manhattan plots for the *ERECTA* phenotype** . (A) shows the results for Linkage analysis (HAPPY software), while (B) shows the results for genome-wide association analysis (GAPIT software). (C) shows the estimated effect of the 19 alleles at the closest marker associated with the linkage analysis peak (MASC05927, chr2:11174979)

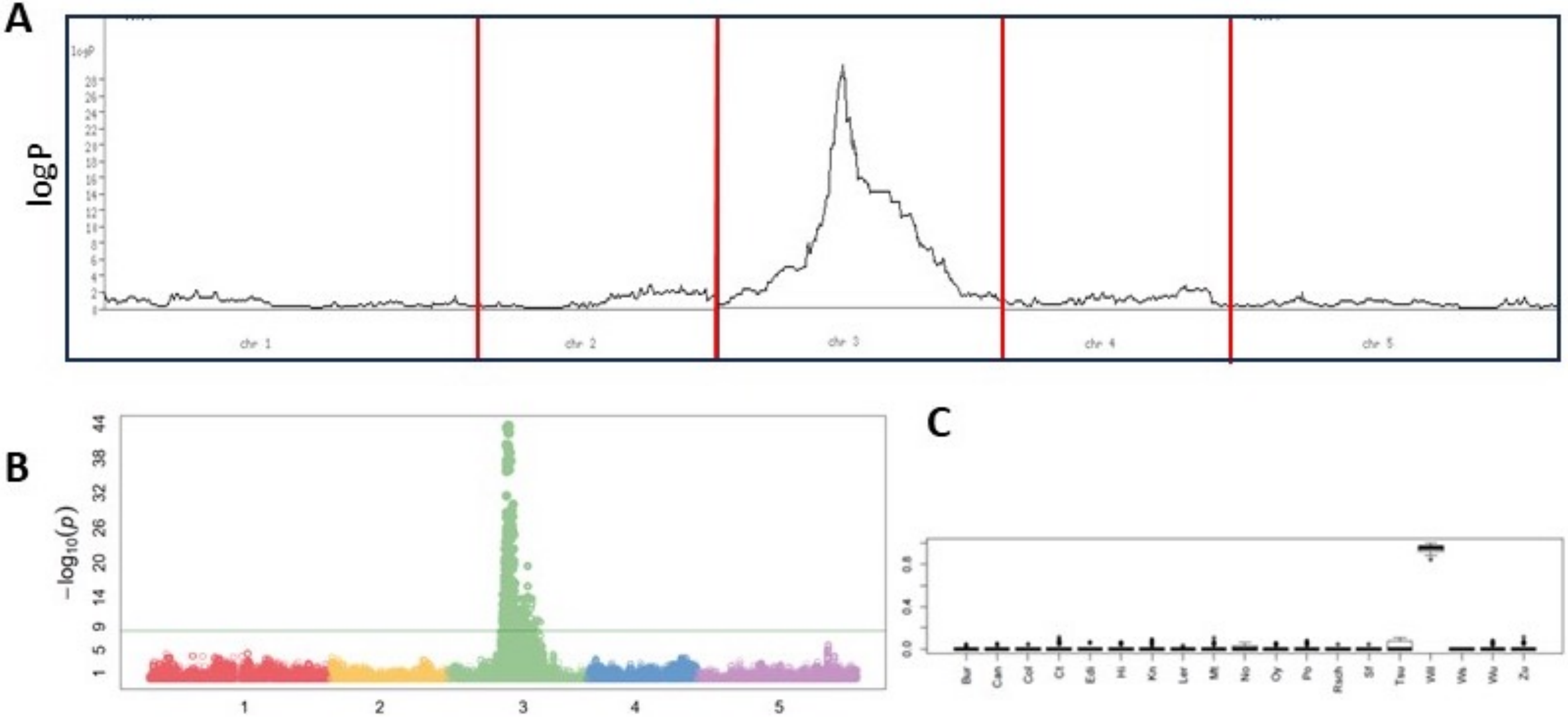

Figure S3. **Plot combining LOD curves and Manhattan plot for the *GLABROUS* phenotype**. (A) shows the results for Linkage analysis (HAPPY software), while (B) shows the results for genome-wide association analysis (GAPIT software). (C) shows the estimated effect of the 19 alleles at the closest marker associated with the linkage analysis peak (MN3_10363610, chr3: 10363610)
